# An approach for differentiating echovirus 30 and Japanese encephalitis virus infections in acute meningitis/encephalitis: a retrospective study of 103 cases in Vietnam

**DOI:** 10.1186/1743-422X-10-280

**Published:** 2013-09-11

**Authors:** Yuki Takamatsu, Leo Uchida, Phan Thi Nga, Kenta Okamoto, Takeshi Nabeshima, Dang Thi Thu Thao, Do Thien Hai, Nguyen Thi Tuyet, Hoang Minh Duc, Le Xuan Luat, Futoshi Hasebe, Kouichi Morita

**Affiliations:** 1 Department of Virology, Institute of Tropical Medicine, Nagasaki University, 1-12-4, Sakamoto, 852-8523 Nagasaki, Japan; 2 Graduate school of Biochemical Sciences, Nagasaki University, Nagasaki, Japan; 3 Department of Training and Research Management, National Institute of Hygiene and Epidemiology, Hanoi, Vietnam; 4 Department of Infectious Disease, National Hospital of Pediatrics, Hanoi, Vietnam; 5 Department of Infectious Disease, Bac Giang General Hospital, Bac Giang, Vietnam; 6 General Department of Preventive Medicine, Ministry of Health, Hanoi, Vietnam

**Keywords:** Echovirus 30, Japanese encephalitis virus, Acute meningitis/encephalitis

## Abstract

**Background:**

In recent decades, Echovirus 30 (E30) and Japanese encephalitis virus (JEV) have been reported to be the common causative agents of acute meningitis among patients in South East Asia. An E30 outbreak in Vietnam in 2001–2002 gained our interest because the initial clinical diagnosis of infected patients was due to JEV infection. There are few clinical insights regarding E30 cases, and there are no reports comparing E30 and JEV acute meningitis/encephalitis cases based on clinical symptoms and case histories. We therefore aimed to identify reliable clinical methods to differentiate E30 and JEV acute meningitis/encephalitis.

**Methods:**

A retrospective, cross-sectional study was conducted to compare E30 and JEV acute meningitis/encephalitis cases. We collected and analyzed the clinical records of 43 E30 confirmed cases (E30 group) and 60 JEV confirmed cases (JEV group). Clinical data were compared between the E30 and the JEV groups. Differences of clinical parameters were analyzed by certain statistical tests.

**Results:**

Fever, headache, and vomiting were the most common symptoms in both the E30 and the JEV groups. Combined symptoms of headache and vomiting and the triad of symptoms of fever, headache, and vomiting were observed in more patients in the E30 group (E30 vs. JEV: 19% vs. 0%, p < 0.001; 74% vs. 27%, p < 0.001, respectively). On the other hand, strong neurological symptoms such as seizure (5% vs. 73%, p < 0.001) and altered consciousness (12% vs. 97%, p < 0.001) were manifested primarily in the JEV group. CSF leukocytosis was observed predominantly in the E30 group (80 vs. 18 cells/μL, p = 0.003), whereas decreasing CSF sugar level was observed predominantly in the JEV group (58.7 vs. 46.9 mg/dL, p < 0.001).

**Conclusion:**

Fever, headache, vomiting, absence of neurological symptoms (seizure, altered consciousness), and presence of CSF leukocytosis are important parameters to consider in differentiating E30 from JEV cases during early infection. Then, proper measures can be adopted immediately to prevent the spread of the disease in the affected areas.

## Background

E30 is a positive-strand RNA virus that belongs to the family *Picornaviridae*, genus *Enterovirus*[1]. It is transmitted to humans by the oral-fecal route [2]. E30 is responsible for many sporadic outbreaks of aseptic meningitis in many countries because of its easier transmission [1-9]. Japanese encephalitis virus (JEV) is a mosquito-borne virus that belongs to family *Flaviviridae*, genus *Flavivirus*[10]. It can cause meningitis or encephalitis in humans. JEV causes approximately 30,000-50,000 meningitis/encephalitis cases annually in Asia, and it is one of the leading causes of the meningitis/encephalitis cases worldwide [10,11]. In recent decades, many E30 outbreaks have occurred in JEV-endemic Asian countries around Vietnam [3,5,12-14]. According to several case reports on meningitis in these countries, an epidemic season of E30 overlapped with that of JEV [5,12,15,16]. Hence, certainty on clinical diagnosis cannot be guaranteed, especially during the early course of illness.

The general symptoms of *Enterovirus* meningitis are headache, nausea, and vomiting. Common cold symptoms are also observed [1,5]. In some instances, severe illness characterized by paralysis and encephalitis leads to death [9,13]. These symptoms varying from mild to severe manifestations are quite similar to those due to JEV infection [11,17,18]. Thus, a correct identification of the causative agent is difficult to determine based on the clinical symptoms.

Several reports showed that JEV is one of the leading causes of acute meningitis/encephalitis in Vietnam [15,16,18]. However, the number of JEV-confirmed cases was not high enough, and some of the patients were found to actually be infected by enteroviruses instead [18,19]. In this report, we consider only those patients whose admitting diagnosis was acute meningitis/encephalitis, and whose infection was confirmed to be due to E30 or JEV by laboratory procedures. Our study focused on the clinical information of these patients, and we found that specific clinical symptoms and laboratory findings could give the clinicians/epidemiologists a more reliable method for differentiating E30 and JEV cases as early as possible.

## Materials and methods

### Ethical statements

This study was approved by the Institutional Review Board of the National Institute of Hygiene and Epidemiology (NIHE), Vietnam (No: 01 IRB, November 7, 2005, No: 33 IRB, December 15, 2011).

### Specimen collection

Cerebrospinal fluid (CSF) specimens were collected only from patients who were clinically diagnosed to have acute meningitis/encephalitis at the time of admission and whose clinical records were available. The patients were from the National Hospital of Pediatrics (NHP) in Hanoi, Northern Vietnam and from Bac Giang General Hospital (BGGH) in Bac Giang, Northern Vietnam. The period of collection at the NHP was from 2001–2002, when an E30 outbreak occurred in Hanoi. The period of collection at BGGH was from 1999 to 2008. NIHE collects clinical specimens from BGGH annually because it is located in a JEV endemic area [16].

### Laboratory investigation

CSF specimens were sent to the NIHE for laboratory diagnosis. The E30 cases were identified by the neutralization test (NT) using anti-E30 serum [20]. Several samples that were unidentified by the NT were subjected to the virus isolation and gene amplification method as described below. The JEV cases were confirmed by IgM Capture ELISA [16].

### Virus isolation, RT-PCR, and sequencing

Eight unidentified samples were subjected to virus isolation. Each CSF specimen was inoculated in human rhabdomyosarcoma cells (RD cells). The cells were incubated at 37°C with 5% CO_2_ until the cytopathic effect (CPE) was observed under a microscope [21,22]. Then, the infected culture fluids (ICFs) were collected and kept at -80°C prior to use. The viral RNA was isolated from the ICFs by the QIAamp Viral RNA Mini Kit (QIAGEN) according to the manufacturer’s instructions [23]. To amplify the complete VP1 gene of E30, RNA templates were subjected to reverse transcription and polymerase chain reaction (RT-PCR) using the forward primer 5′-GCRTGCAATGAYTTCTCWGT-3′ and the reverse primer 5′-GCICCIGAYTGITGICCRAA-3′ [24]. The amplicons were sequenced using the ABI PRISM 3100-*Avant* Genetic Analyzer [25].

### Phylogenetic analysis

**Figure 1:**
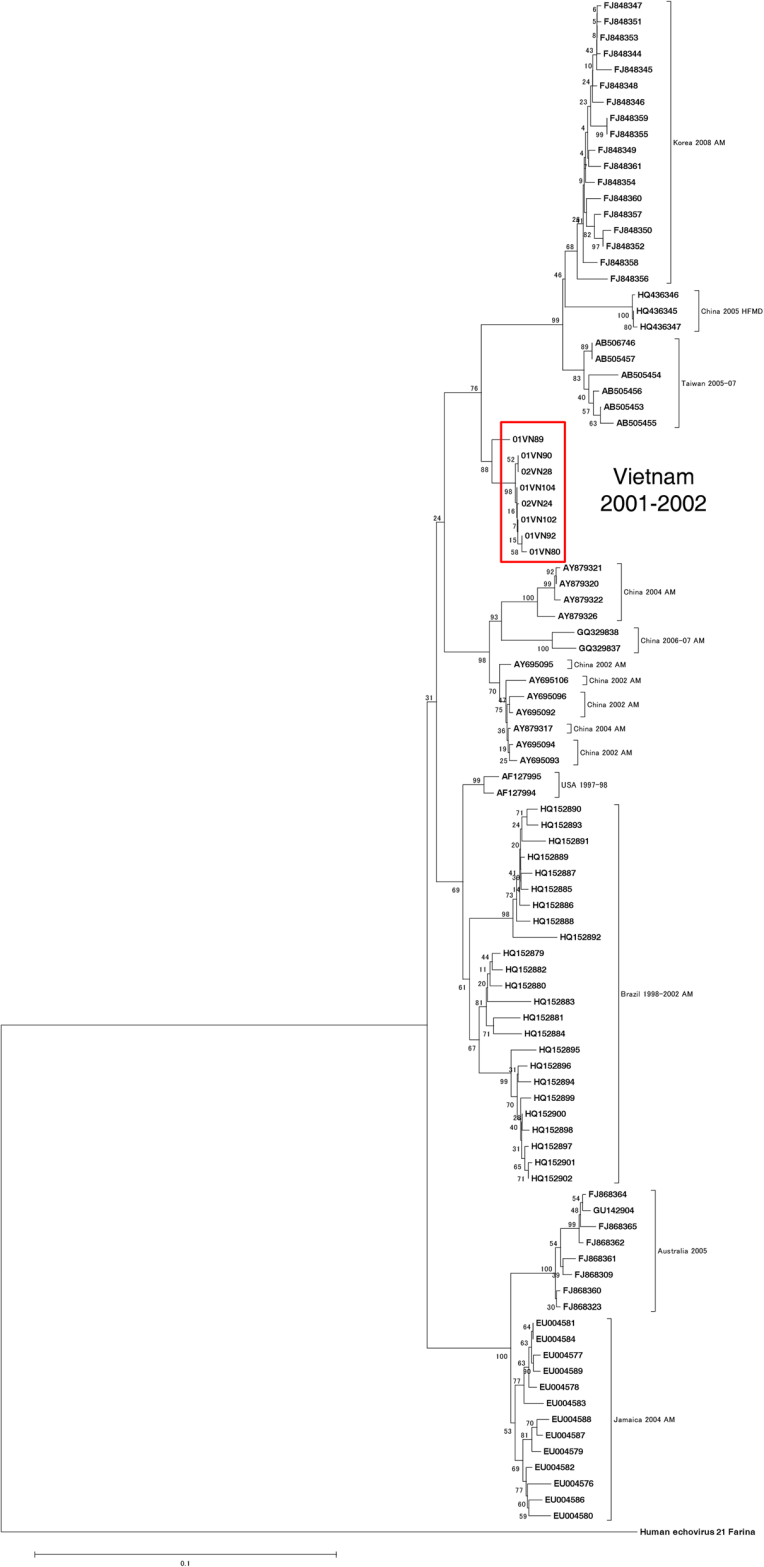
**Phylogenetic analysis of the selected E30 strains.** A phylogenetic analysis of VP1 gene sequences from the selected human E30 strains from different geographical origins was conducted. The neighbor-joining tree was generated using MEGA 5.0 software, and the prototype strain Farina of echovirus 21 was used as the outgroup. The eight Vietnamese isolates are highlighted in the red colored box. The sequences reported here have been deposited in GenBank with the accession numbers of KC999616 to KC999623.

Phylogenetic analysis of selected strains of human E30 from different geographical origins was performed based on the VP1 gene sequences (Figure 1). Alignment of these sequences was performed by Clustal W version 2.0 [26], and a neighbor-joining tree [27] was generated using MEGA 5.0 software [28]. The prototype strain Farina of Echovirus 21 (GenBank accession number: AY302547) was used as the out-group. The reliability of the phylogenetic tree was determined by a bootstrap resampling test with 1,000 replicates.

### Clinical data and statistical analysis

The clinical data from E30-confirmed cases (E30 group) and JEV-confirmed cases (JEV group) were included in the statistical analysis. The statistical analysis was conducted using R version 2.15 software [29]. The difference between the E30 and the JEV groups was tested by Chi-squared tests and Fisher Exact tests. The distribution of values between these groups was compared by the F test, and the difference of values between them was assessed by Student’s t-tests or Welch tests. A P value of less than 0.05 was accepted as significant.

## Results

### Laboratory confirmation of E30 and JEV infection

**Figure 2:**
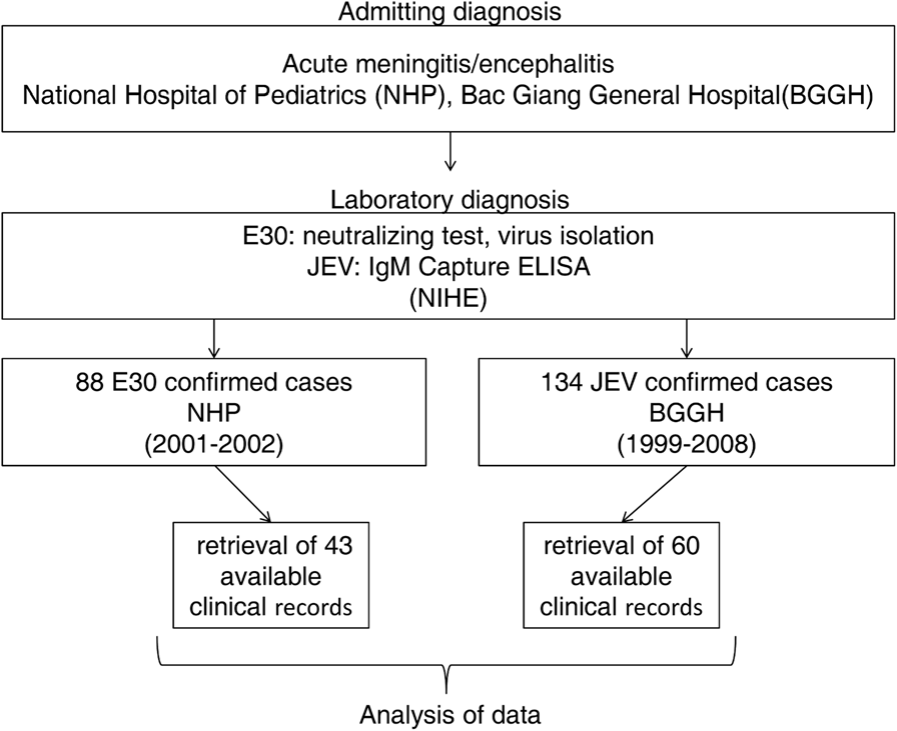
**Flow-chart of the work and some of the results in this study.** CSF specimens were collected from patients who were clinically diagnosed with acute meningitis/encephalitis at the time of admission to the National Hospital of Pediatrics (NHP) during 2001–2001 and Bac Giang General Hospital (BGGH) during 1999–2008. CSF specimens were sent to NIHE for laboratory diagnosis. A total of 88 patients from NHP were confirmed to be infected with E30 based on NT (80 patients) and virus isolation (8 patients) but only 43 patients had available clinical records. From BGGH, a total of 134 patients were confirmed to be infected with JEV based on the IgM Capture ELISA but only 60 had clinical records.

Eighty-eight patients from NHP were confirmed to have E30 infection based on NT (80 patients) and virus isolation (8 patients) (Figure 2). One hundred thirty-four patients at BGGH were confirmed to have JEV infection by IgM Capture ELISA (Figure 2).

### Virus isolation, sequence comparison, and phylogenetic analysis

E30 was isolated from eight CSF specimens. The VP1 gene in these isolates was sequenced. The designated strain name of each isolate and the corresponding GenBank accession number (enclosed in parentheses below) were as follows: 01VN80 (KC999616), 01VN89 (KC999617), 01VN90 (KC999618), 01VN92 (KC999619), 01VN102 (KC999620), 01VN104 (KC999621), 02VN24 (KC999622), and 02VN28 (KC999623).

We constructed the NJ tree of these eight Vietnamese strains together with the 88 E30 strains from GenBank based on the VP1 gene sequence. Based on this tree, our identified strains were relatively close to the Asian strains that caused aseptic meningitis (Figure 1).

### Epidemiological features

**Figure 3:**
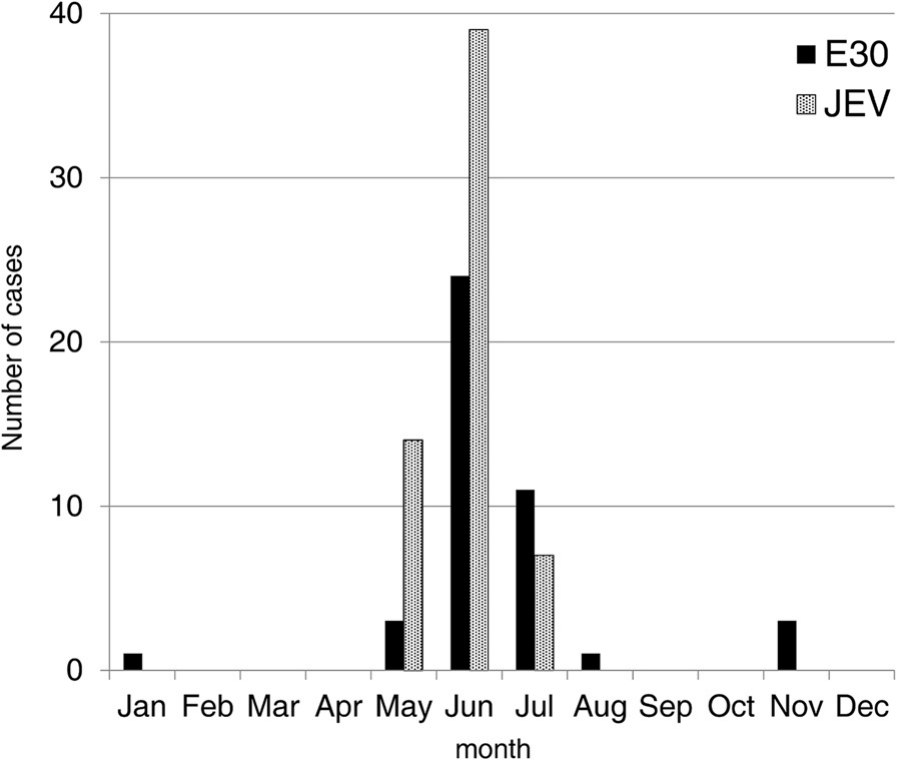
**Monthly distribution and frequency of E30 and JEV cases.** There were 43 confirmed E30 cases from NHP during 2001–2002 and 60 JEV confirmed cases from BGGH during 1999–2008. The E30 cases and the JEV cases were totaled separately according to the month and regardless of the year of occurrence. The total number of cases per month from January to December was plotted in the graph. The peak for both cases was from May to July.

Most patients were under 15 years old in both the E30 and the JEV groups. The median age was 7 in the former group and 6 in the latter group. In the E30 group, there were 30 male patients (70%) and 13 female patients (30%), with a male-to-female ratio of 2.3:1. In the JEV group, there were 22 male patients (37%) and 38 female patients (63%), with a male-to-female ratio of 1:1.7. The percentage of JEV-vaccinated patients in the E30 group was significantly higher than that in the JEV group (E30 vs. JEV: 56% vs. 12%, p < 0.001) (Table 1). Fatal (12%) and sequelae (10%) cases were observed in the JEV group only (Table 1). Both E30 and JEV cases occurred more frequently during the summer season from May to July (Figure 3).

**Table 1:** Basic patient information and outcome

		**Median age [range] or number of patients (%)**		
		**E30 patients**	**JEV patients**	**p-value**
		**n = 43**	**n = 60**	
Age		7 [0.4-14]	6 [0.8-21]	0.916
Sex	Male	30 (70)	22 (37)	
	Female	13 (30)	38 (63)	
Family/neighbor history^1^		1 (2)	1 (2)	0.628
JEV vaccination		24 (56)	7 (12)	<0.001*
Death		0 (0)	7 (12)	0.040*
Sequelae		0 (0)	6 (10)	0.039*

^1^family or neighbor showed similar symptoms as the patient at that time, *significant difference (p < 0.05).

### Clinical features during admission

The clinical features during admission of the E30 and the JEV patients are shown in Table 2. The vital sign parameters (body temperature, pulse rate, and respiratory rate) on admission day showed no significant difference between the two groups of patients. A longer hospitalization period was observed in the JEV group than in the E30 group (E30 vs. JEV: 7 [range: 3–23] vs. 9 [1–37] days, p = 0.003). More patients in the E30 group complained of headache (95% vs. 50%, p < 0.001), vomiting (98% vs. 33%, p < 0.001), abdominal symptoms (19% vs. 5%, p = 0.048), and neck or back pain (26% vs. 2%, p < 0.001). On the other hand, more patients in the JEV group had a higher maximum body temperature (38.0 [37.0-41.0] vs. 38.8 [37.0-40.0] °C, p = 0.010), altered consciousness (12% vs. 97%, p < 0.001), and seizures (5% vs. 73%, p < 0.001). The periods of altered consciousness (1 [1-3] vs. 3 [1-17] days, p = 0.002), seizures (1 [1] vs. 2 [1-7] days, p < 0.001), Babinski reflex (1 [1-3] vs. 2 [1-7] days, p = 0.001), and focal neurological signs (1 [1] vs. 2.5 [1-13] days, p = 0.037) lasted longer in the JEV group. Both groups had a similar percentage of patients with signs of meningeal irritations, whereas the duration period was longer in the JEV group (1 [1-6] vs. 3 [1-14] days, p = 0.002). More patients in the JEV group experienced Babinski reflex (42% vs. 62%, p = 0.047), and with a longer duration (1 [1-3] vs. 2 [1-7] days, p = 0.001). Hemiplegia was observed only in the JEV group (25%, p < 0.001).

**Table 2:** Clinical features during admission

**Clinical feature(s)**	**Median [range] or, number of patients with the feature (%)**		**p-value**
	**E30 patients**	**JEV patients**	
	**n = 43**	**n = 60**	
Body temperature (°C)^1^	38.0 [36.2-41.0]	38.5 [36.5-40.0]	0.096
Pulse rate (beats per min.)^1^	100 [80–120] {34}^2^	100 [70–145] {58}^2^	0.092
Respiratory rate (breaths per min.)^1^	26 [20–45] {13}^2^	26 [20–50] {31}^2^	0.691
Length of stay in the hospital (day)	7 [3-23]	9 [1–37]	0.003*
Highest body temperature (°C)	38.0 [37.0-41.0]	38.8 [37.0-40.0]	0.010*
Period of fever^3^ (day)	1 [1-14]	2 [1-7]	0.259
Headache	41 (95)	30 (50)	< 0.001*
Period of headache (day)	2 [1-7] {41}	2.5 [1-12] {30}	0.006*
Vomiting	42 (98)	20 (33)	< 0.001*
Period of vomiting (day)	1 [1-5] {42}	1 [1,2] {20}	0.526
Altered consciousness	5 (12)	58 (97)	< 0.001*
Period of altered consciousness (day)	1 [1-3] {5}	3 [1-17] {58}	0.002*
Seizure	2 (5)	44 (73)	< 0.001*
Period of seizure (day)	1 [1] {2}	2 [1-7] {44}	< 0.001*
Sign of meningeal irritation	30 (70)	41 (68)	0.877
Period of meningeal irritation (day)	1 [1-6] {30}	3 [1-14] {41}	0.002*
Babinski reflex	18 (42)	37 (62)	0.047*
Period of Babinski reflex (day)	1 [1-3] {18}	2 [1-7] {37}	0.001*
Focal neurological sign	2 (5)	10 (17)	0.070
Period of focal neurological sign (day)	1 [1] {2}	2.5 [1-13] {10}	0.037*
Dyspnea	2 (5)	10 (17)	0.070
Hemiplegia	0 (0)	15 (25)	< 0.001*
Fatigue	14 (33)	22 (37)	0.666
Abdominal symptom	8 (19)	3 (5)	0.048*
Neck or back pain	11 (26)	1 (2)	< 0.001*
Joint pain	1 (2)	0 (0)	0.235
Skin rash	0 (0)	1 (2)	0.395
Fever^3^ + Headache	0 (0)	11 (18)	0.002*
Headache + Vomiting	8 (19)	0 (0)	< 0.001*
Vomiting + Fever^3^	4 (9)	2 (3)	0.232
Fever^3^ + Headache + Vomiting	32 (74)	16 (27)	< 0.001*

^1^taken on the day of admission, ^2^number inside curly brackets refer to the number of patients with the record, ^3^fever defined as body temperature of over 38°C, *significant difference (p < 0.05).

We also focused on the symptoms observed simultaneously. The combined symptoms of headache and vomiting and the triad of symptoms of fever, headache, and vomiting were observed in more patients in the E30 group (19% vs. 0%, p < 0.001 and 74% vs. 27%, p < 0.001, respectively). On the other hand, occurrence of the combined symptoms of fever and headache was observed only in the JEV group (18%, p = 0.002).

### Laboratory findings

The results of the CSF and blood examination are shown in Table 3. CSF leucocytes were significantly increased in the E30 group (80 [1–750] vs. 18 [5–350] cells/μL, p = 0.003). An increased number of both CSF neutrophils and CSF lymphocytes was observed in the E30 group (neutrophils: 26 [2–381] vs. 10 [3–140] cells/μL, p = 0.033, lymphocytes: 56 [3–676] vs. 12 [4–210] cells/μL, p = 0.043). The CSF sugar levels were significantly decreased in the JEV group (58.7 [27–90] vs. 46.9 [25–74] mg/dL, p < 0.001). Other indicators of CSF and blood components showed no significant difference between the E30 and the JEV groups.

**Table 3:** CSF and blood examination

	**Median value (range), [number of patients]**		**p-value**
	**E30 patients**	**JEV patients**	
	**n = 43**	**n = 60**	
CSF protein (mg/dL)	36.3 (14–144) [39]	44.5 (6–100) [47]	0.093
CSF sugar (mg/dL)	58.7 (27–90) [38]	46.9 (25–74) [47]	< 0.001*
CSF leucocyte (cells/μL)	80 (1–750) [41]	18 (5–350) [45]	0.003*
CSF neutrophil (cells/μL)	26 (2–381) [23]	10 (3–140) [35]	0.033*
CSF lymphocyte (cells/μL)	56 (3–676) [23]	12 (4–210) [35]	0.043*
Blood leucocyte (10E3 cells/μL)	10.4 (5.8-34) [19]	12.0 (3.0-30) [59]	0.711
Blood neutrophil (10E3 cells/μL)	8.2 (3.9-25) [18]	8.0 (1.1-26) [57]	0.368
Blood lymphocyte (10E3 cells/μL)	2.1 (1.3-9.6) [18]	3.2 (1.3-12) [57]	0.189

*significant difference (p < 0.05).

### Treatment features

The laboratory diagnosis required at least one week to complete, thus medication was given even before the results of the diagnosis became available. The medication histories of the E30 and the JEV groups are shown in Table 4. A history of the use of steroids showed no significant difference between the two groups (E30 vs. JEV: 98% vs. 97%). The duration of steroids use also showed no difference in both groups (3 [2-4] vs. 3 [2-15] days). More patients were prescribed mannitol (47% vs. 95%, p < 0.001) and antibiotics (35% vs. 95%, p < 0.001) in the JEV group. The duration of mannitol use was longer in the JEV group (1 [1-3] vs. 3 [1-7] days, p < 0.001). A history of the use of diazepam showed no significant difference between the two groups. The duration of diazepam use was longer in the E30 group (6 [2-14] vs. 2 [1-11] days, p < 0.001). More patients in the JEV group were prescribed barbiturates (33% vs. 63%, p = 0.002), chlorpromazine (5% vs. 23%, p = 0.012), fentanyl (0% vs. 33%, p < 0.001), and sodium valproate (0% vs. 33%, p < 0.001).

**Table 4:** Treatment features

	**Number of patients (%), median day [range]**		**p-value**
	**E30 patients**	**JEV patients**	
	**n = 43**	**n = 60+**	
Steroids	42 (98)	58 (97)	0.769
Period of treatment	3 [2-4]	3 [2-15]	0.204
Mannitol	20 (47)	57 (95)	< 0.001*
Period of treatment	1 [1-3]	3 [1-7]	< 0.001*
Antibiotics	15 (35)	57 (95)	< 0.001*
Period of treatment	8 [1-15]	8 [1-17]	0.688
Diazepam	23 (53)	32 (53)	0.988
Period of treatment	6 [2-14]	2 [1-11]	< 0.001*
Barbiturate	14 (33)	38 (63)	0.002*
Period of treatment	3.5 [1-14]	2.5 [1-11]	0.130
Chlorpromazine	2 (5)	14 (23)	0.012*
Fentanyl	0 (0)	20 (33)	< 0.001*
Sodium Valproate	0 (0)	20 (33)	< 0.001*

*significant difference (p < 0.05).

## Discussion

E30 and JEV are the causative agents of acute meningitis/encephalitis in Asian countries. In the present report, Vietnamese patients with an admitting diagnosis of acute meningitis/encephalitis were confirmed to have either E30 or JEV infection by a laboratory diagnosis. The E30 CSF samples analyzed in the present study were collected during the outbreak in 2001–2002. Eight E30 strains were isolated from this outbreak, and the phylogenetic analysis showed that these are relatively close to the strains isolated during E30 outbreaks from other Asian countries (Figure 1).

The E30 acute meningitis/encephalitis outbreak in 2001–2002 gained our interest because nearly all of the patients were clinically diagnosed to have Japanese encephalitis at the beginning of their hospitalization. The reason for this initial diagnosis could be the lack of clear guidelines for differentiating E30 from JEV infections based on the clinical symptoms and case histories. Our study is the first report to show that certain clinical features could differentiate E30 from JEV cases.

We tried to compare the symptoms and clinical features between the two groups before admission, according to the information in the clinical record (unpublished data). However, there was no sufficient difference noted between the E30 and the JEV cases compared to those during admission (Table 2). Then, we focused our attention on the data during the admission period. Here, we noted that the most common symptoms were fever, headache, and vomiting for both the E30 and the JEV groups (Table 2). More patients in the E30 group had the combined symptoms of headache and vomiting and the triad of symptoms (fever, headache, and vomiting) (Table 2). Thus, it is concluded that these combined symptoms could indicate a high probability of E30 infection. Findings on magnetic resonance imaging (MRI) of patients in the E30 cases revealed no significant abnormalities in the brain [7]. It seems that the mild neurological symptoms observed in the E30 group are not from direct neuronal damage, but from increased intracranial pressure. Therefore, vomiting, which is one of the important signs of increased intracranial pressure, was often observed in the E30 group compared to the JEV group in this study.

More patients had altered consciousness (97%), seizure (73%), and hemiplegia (25%) in the JEV group (Table 2). These symptoms are strong signs of neural pathogenicity. It was reported that the JEV patients showed abnormalities in thalamus, basal ganglia, and cerebral cortex based on the results of computed tomography (CT) and MRI [30-32]. However, it is difficult to conduct these expensive examinations in a resource limiting setting in the rural areas of Asian countries. Neuropathological findings in fatal JEV cases showed that the virus caused broad inflammation in the central nervous system [33]. JEV infects neurons and causes direct damage [10,33] that consequently leads to many severe neurological symptoms. Thus, in areas where E30 and JEV outbreaks are overlapping, severe neurological symptoms could provide strong evidence of JEV infection.

The most critical laboratory finding in this report was the CSF leukocytosis in the E30 group (Table 3). An increase in CSF neutrophils was observed, and this was also reported in another study [34]. An increase in CSF lymphocytes was observed in the present study. Therefore, these results seem to suggest that CSF leukocytosis relates to the E30 infection.

Based on the medication history, more patients were prescribed barbiturates, chlorpromazine, fentanyl, and sodium valproate in the JEV group (Table 4). This seems to correlate with the longer period of neurological symptoms such as altered consciousness and seizures in the JEV group. We would like to indicate that medication strategies have been established in each hospital, however some minor differences in these strategies exist between hospitals.

Patients in the E30 group had a higher male-to-female ratio of 2.3:1. A higher ratio was also shown in previous reports [34,35]. Boys usually prefer to play outside of their house compared with girls. This behavior could result in a more frequent exposure of boys to the pathogen.

## Conclusion

Clinical information demonstrated that the combination of headache and vomiting and the triad of symptoms of fever, headache, and vomiting could indicate a high probability of E30 infection. A laboratory parameter of CSF leukocytosis also suggests E30 infection. On the other hand, strong neurological symptoms, such as altered consciousness, seizure, and hemiplegia, could indicate a high probability of JEV infection. These findings will help improve the clinical diagnosis of patients with E30 or JEV meningitis/encephalitis. Then, proper measures can be adopted for the clinical management of the patients at the early phase of admission before laboratory diagnosis becomes available.

## Abbreviations

E30: Echovirus 30; JEV: Japanese encephalitis virus; CSF: Cerebrospinal fluid; NIHE: National Institute of Hygiene and Epidemiology; NT: Neutralization test; RD cells: Rhabdomyosarcoma cells; ICF: Infected Culture Fluid; CPE: Cytopathic effect; RT-PCR: Reverse transcription and polymerase chain reaction.

## Competing interests

The authors declare that they have no competing interests.

## Authors’ contributions

DTH, NTT, HMD, and PTN contributed to the collection of the specimens and clinical diagnosis. NTP and FH contributed to the laboratory diagnosis. YT, LU, KO, and TN performed the genome analysis and cell culture. YT, DTT, PTN, and FH collected clinical information and analyzed these data. YT and KM designed the study. YT and LXL drafted the manuscript. All of the authors read and approved the final version of the manuscript.
